# COVID-19 Pandemic Impact on Respiratory Infectious Diseases in Primary Care Practice in Children

**DOI:** 10.3389/fped.2021.722483

**Published:** 2021-09-13

**Authors:** Ravinder Kaur, Steven Schulz, Naoko Fuji, Michael Pichichero

**Affiliations:** Center for Infectious Diseases and Immunology, Rochester General Hospital Research Institute, Rochester, NY, United States

**Keywords:** infections during COVID-19, nasopharyngeal colonization, respiratory infections, acute otitis media, antibiotics

## Abstract

**Background:** The coronavirus disease 2019 (COVID-19) pandemic led to day care and school closures and children staying home for several months. When they gradually returned, aggressive regulations were implemented in New York State to reduce viral transmission.

**Method:** An ongoing prospective study occurring in the Rochester, NY region, focused on early childhood respiratory infectious diseases, afforded an opportunity to assess the impact of the pandemic on the incidence of these illnesses in a primary care outpatient setting. Physician-diagnosed, medically attended infection visits were assessed in two child cohorts, age 6–36 months old: from March 15 to December 31, 2020 (the pandemic period) compared to the same months in 2019 (prepandemic). Nasopharyngeal colonization by potential otopathogens during healthy/well-child and acute otitis media (AOM) visits was evaluated.

**Results:** One hundred and forty-four children were included in the pandemic cohort and 215 in the prepandemic cohort. The pandemic cohort of children experienced 1.8-fold less frequent infectious disease visits during the pandemic (*p* < 0.0001). Specifically, visits for AOM were 3.7-fold lower (*p* < 0.0001), viral upper respiratory infections (URI) 3.8-fold lower (*p* < 0.0001), croup 27.5-fold lower (*p* < 0.0001), and bronchiolitis 7.4-fold lower (*p* = 0.04) than the prepandemic cohort. *Streptococcus pneumoniae* (*p* = 0.03), *Haemophilus influenzae* (*p* < 0.0001), and *Moraxella catarrhalis* (*p* < 0.0001) nasopharyngeal colonization occurred less frequently among children during the pandemic.

**Conclusion:** In primary care pediatric practice, during the first 9 months of the COVID-19 pandemic, significant decreases in the frequency of multiple respiratory infections and nasopharyngeal colonization by potential bacterial respiratory pathogens occurred in children age 6–36 months old.

## Introduction

On March 12, 2020, a public health emergency was declared in the United States (U.S.) as a response to the severe acute respiratory syndrome coronavirus 2 (SARS-CoV-2) pandemic. Closure of schools and day care facilities and an order to quarantine at home were enacted in New York State to reduce or prevent the transmission of SARS-CoV-2 infection and the disease known as coronavirus disease 2019 (COVID-19) (1). Similar restrictions were imposed in other States and other countries (2–5). In this rare event of a worldwide pandemic, public health actions implemented were effective to control the spread of SARS-CoV-2 infections (6).

A secondary consequence of public health measures to prevent the spread of SARS-CoV-2 included a concurrent reduction in risk for children to acquire and spread other respiratory viral infectious disease illnesses. An ongoing prospective study in primary care pediatric practices in the Rochester, NY area, afforded an opportunity to assess the impact of the pandemic control measures on infectious disease illness visits in young children. Specifically, in children aged 6–36 months old, a study partly supported by the U.S. Centers for Disease and Prevention (CDC) was in place when the pandemic began with a primary objective to evaluate the changing epidemiology of acute otitis media (AOM) and nasopharyngeal (NP) colonization by potential bacterial respiratory pathogens in two community-based primary care pediatric practices. As the public health measures mandated by NY State Department of Health were implemented, we prospectively quantified their impact on physician-diagnosed infectious disease illness visits. Here, we report the incidence of infectious disease visits by a cohort of young children during the COVID-19 pandemic period March 15, 2020, through December 31, 2020 compared to the same time frame in the preceding year, 2019 in Rochester, NY.

## Methods

### Study Population, Setting, and Design

The study children were participating in an ongoing prospective, longitudinal study with a primary objective to assess the incidence and dynamic changes in etiology of AOM and NP colonization by potential respiratory bacterial pathogens over time. The study design called for the collection of NP samples at scheduled healthy/well-child visits at age 6, 9, 12, 15, 18, 24, and 36 months and middle ear fluid by tympanocentesis when children experienced AOM. All the study children received their primary care from two participating hospital-affiliated pediatric clinical practices within the Rochester, NY region (Bay Creek Pediatrics, Webster, NY, a suburb of Rochester in Monroe County, and Finger Lakes Medical Associates Pediatrics, Geneva, NY, in Ontario County). The practices were comprised of a typical mixed demographic of lower and middle class, healthcare-insured families, including public assistance insurance for some. All children received routine pediatric vaccinations according to the schedule recommended by the CDC. The study design included capturing every physician-diagnosed, medically attended infectious disease illness. Demographic and infection risk factor data were collected including family history of AOM, daycare attendance, breastfeeding, and tobacco smoke exposure.

For the secondary objective of assessing the incidence of all respiratory and other infections among 6- to 36-month-old children, the electronic medical record (EMR) was used to capture and record such visits to the primary care site as well as all virtual visits and phone contacts and to identify all visits made by study children to other healthcare facilities, i.e., urgent care and hospital emergency rooms. Infectious diseases often have seasonal variation; therefore, infection visit incidence was assessed from March 15 to December 31, 2020 (beginning of pandemic) and compared with children during the same months in 2019 (prepandemic) in the same pediatric practice sites. Written informed consent was obtained from parents at enrollment in the study as approved by the Rochester Regional Health Institutional Review Board.

### Definition of AOM

AOM was diagnosed by pneumatic otoscopy by validated otoscopists according to American Academy of Pediatrics guidelines (7). Episodes of clinically diagnosed AOM were confirmed by culture of middle ear fluid (MEF) collected by tympanocentesis up to 36 months of age in the prepandemic but not in the pandemic cohort (discussed below). We have previously reported that tympanocentesis cultures collected from our study cohort yield *Streptococcus pneumoniae, Haemophilus influenzae*, or *Moraxella catarrhalis* by culture or polymerase chain reaction (PCR) in ~90% of MEF samples (8, 9).

### Other Respiratory Infection Visit Definitions

Viral upper respiratory infections (URIs) were diagnosed clinically as previously described (10). We have previously reported that viral URI clinical diagnosis in our study cohort is confirmed by PCR in 66% of NP samples (11, 12). Lobar pneumonia was diagnosed based on the presence of fever, increased respiratory rate for age, and examination revealing lobar consolidation. Chest radiographs were not obtained, in keeping with the guidelines of the Infectious Diseases Society of America (13). Acute bacterial rhinosinusitis was diagnosed consistent with the American Academy of Pediatrics guidelines (14). Influenza infection was diagnosed clinically and etiology by detection of the virus in NP swab. Other respiratory infections were clinically diagnosed.

### Recommendations of the NY State Health Department for Public Health During the Study Time Frame

On March 7, 2020, a state of emergency in NY due to the COVID-19 pandemic was declared (1). The first patient to test positive for SARS-CoV-2 in Monroe County, NY was on March 11, 2020 (15). All schools were required to close by March 22, 2020 (16). A mandated public use of masks order for adults and children >2 years of age was enacted on April 17, 2020 (17). In the Finger Lakes Region of Upstate NY, where the two primary care pediatric practices reside, complete lockdown was partially lifted on May 15, 2020, and further lifted on June 26, 2020 in accordance with the Un-PAUSE NY Blue Print Plan (18). Almost all regional school districts, including those of the Bay Creek and FLMA Pediatrics practice areas, opened to at least hybrid learning models with many reopening fully for all students starting September 8, 2020. Some districts in the Bay Creek and FLMA Pediatrics practice areas underwent periodic school lockdowns with all virtual learning based on positive test results in schools. Further details are provided in the Supplementary Material.

### Modifications in Medical Care Due to the Pandemic

On March 6, 2020, video telehealth and telephone call visits were introduced as routine practice. Well visits were limited to <2 years of age, then gradually expanded to all ages by late May 2020. Further details are provided in the Supplementary Material.

### Changed Practices in Day Care and Schools

During the “stay at home” phase of the NY State lockdown, day care services were considered an essential business (19). Day care child density was limited (20).

All children >2 years old were required to wear mask while in the facility. Upon arrival, children with any respiratory symptoms or fever were excluded (21, 22). For the school year commencing September 2020, almost all regional school districts, including those of the Bay Creek and FLMA Pediatrics practice areas, opened to hybrid learning models. Exclusion occurred similar to the day care facilities. Further details are provided in the Supplementary Material.

### SARS-CoV-2 Testing

The NYS DOH return to school guidelines required SARS-CoV2 testing for any child who screened positive for any symptom of SARS-CoV-2 (fever, sore throat, runny nose, congestion, cough, nausea/vomiting, diarrhea, abdominal pain, headache, fatigue, myalgia, loss of taste or smell, or rash). Testing was available for Bay Creek and FLMA Pediatrics at the beginning of school reopening in early September.

### Microbiology

Standard microbiology processing and identification techniques were used in detecting *Streptococcus pneumoniae* (*Spn*), *Haemophilus influenzae* (*Hflu*), and *Moraxella catarrhalis* (*Mcat*) in NP and MEF samples, as previously described (9, 23). Oxacillin susceptibility test for *Spn* and β-lactamase testing for *Hflu* and *Mcat* were conducted on isolated strains.

### Statistics

Fisher's exact test was used to determine significant differences between the two cohorts. *p*-values are two-tailed, and *p* < 0.05 was used to define statistical significance using GraphPad Prism software, version 6.

## Results

Clinical diagnoses and healthy or well-child visits of 144 children from March 15 to December 31, 2020 (beginning of pandemic) were compared with 215 children during the same months in 2019 (prepandemic). Supplementary Table 1 shows the demographic and respiratory infection risk factors present in the two cohorts. No statistical differences were found.

### SARS-CoV-2 Testing

Pediatric SARS-CoV-2 positivity rates trended up alongside community spread. FLMA Pediatrics had a positivity rate of 1.9% (5/260) in October 2020 and 11.5% (23/200) in December 2020. Bay Creek Pediatrics had a positivity rate of 0% (0/168) in October 2020 and 19% (35/184) in December 2020. Further details of SARS-CoV-2 infection rates in Upstate NY are provided in the Supplementary Material.

### Incidence of Respiratory Infectious Disease Illnesses

Table 1 shows the incidence of infectious disease illness visits in the two study cohorts. The child population of the two clinics was 12,512 children. During the pandemic, 258 infection visits occurred among 144 pandemic cohort children compared with 687 visits among 215 prepandemic cohort children, a 1.8-fold decrease (*p* < 0.0001). The proportion of children with visits for AOM (3.7-fold, *p* < 0.0001), bronchiolitis (7.4-fold, *p* = 0.036), croup (27.5-fold, *p* < 0.0001), and viral URI (3.8-fold, *p* < 0.0001) decreased significantly. Fever without a source (1.4-fold, *p* = 0.009) and skin/soft tissue infection (2.1-fold, *p* = 0.042) represented a higher proportion of visits during the pandemic.

**Table 1:** Comparison of children with all physician-diagnosed, medically attended infectious disease visits in children before and during the COVID-19 pandemic.

**Diagnosis**	**Prepandemic**	**%**	**During pandemic**	**%**	***p*-value**
	**March 15–December 31, 2019**		**March 15–December 31, 2020**		
Total infectious disease visits	687		258		**<0.0001**
Office	510	74.2	152	58.9	<0.0001
Urgent care/ER	128	18.6	31	12	<0.015
Call/treat at home	49	7.1	46	17.8	<0.0001
Virtual	0	0	29	11.2	<0.0001
Total children in cohort	215		144		
Proportion of infectious disease visits	3.2		1.8		**<0.0001**
All AOM	255	37.1	46	17.8	**<0.0001**
AOM with fever	139	20.2	23	8.9	**<0.0001**
AOM without fever	116	16.9	23	8.9	**0.0008**
Bronchiolitis	22	3.2	2	0.8	**0.036**
Conjunctivitis	47	6.8	12	4.7	0.23
Croup	41	6.0	1	0.4	**<0.0001**
Fever without a source	118	17.2	64	24.8	**0.0095**
Influenza	7	1.0	2	0.8	1
Gastroenteritis	60	8.7	25	9.7	0.701
OME/serous OM	56	8.2	18	7.0	0.49
Other infection	14	2.0	5	1.9	1
Pneumonia lobar	11	1.6	1	0.4	0.196
Pharyngitis	25	3.6	14	5.4	0.269
Sinusitis	8	1.2	0	0.0	0.115
Skin/soft tissue infection	18	2.6	14	5.4	**0.042**
Viral URI	342	49.8	60	23.3	**<0.0001**
URI with fever	197	28.7	47	18.2	**0.001**
URI with viral rash	51	7.4	17	6.6	0.78

*Other infections included one to two cases of the following diseases: hand foot mouth, Lyme disease, lymphadenopathy, aphthous stomatitis, thrush, cervical adenitis, tick bite, viral warts*.

*If a child had fever and/or viral URI along with AOM, then the child was not counted in viral URI and fever categories. If a child had fever with a diagnosed infection, such as lobar pneumonia, then fever was not counted separately. If a child had two distinct infectious diseases such as AOM and lobar pneumonia, then both diagnoses were tabulated*.

*P-values marked with bold indicate statistically significance*.

**Figure 1:**
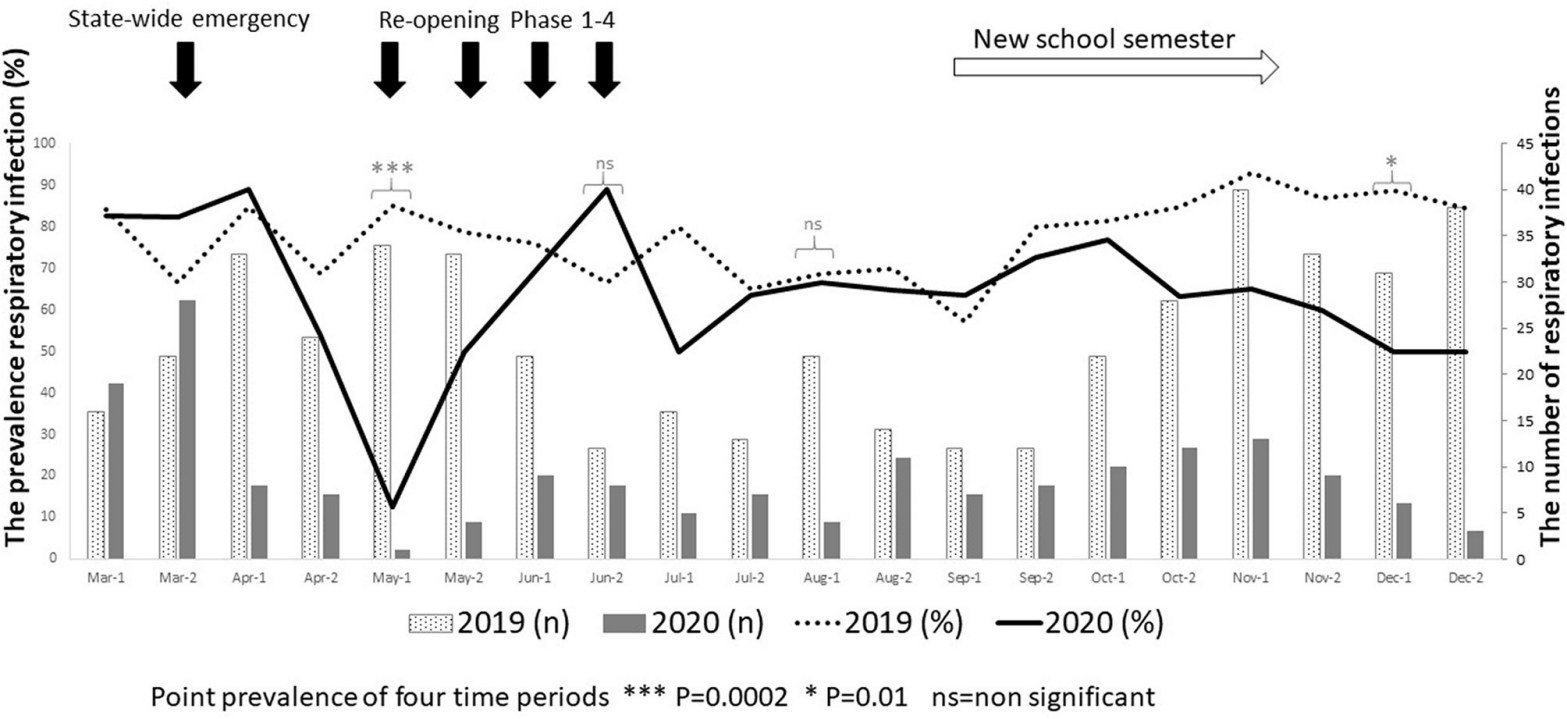
The prevalence of respiratory infections in prepandemic (March–December 2019) and during the pandemic (March–December 2020) in children age “6–30 months.” The left *Y*-axis shows the percentage of respiratory infections, while the right *Y*-axis represents the number of respiratory infections observed in the two times. Fisher's exact test was used to analyze for point prevalence of four time periods.

Figure 1 shows the prevalence of all respiratory infections from March to December in prepandemic and during pandemic time frames. The point prevalence was significantly lower in April 2020 and December 2020 (*p* < 0.05) in the pandemic time frame.

Prescription of antibiotics significantly decreased (*p* < 0.001) during the pandemic. See Supplementary Table 2 for details.

### Change in Care Practices

Possible confounding of the illness visit results could occur due to changes in the frequency of well-child visits and/or changes in the patterns of care from office visits to video telehealth, telephone visits, and referral to Urgent Care and Hospital Emergency Rooms for care. Therefore, we collected data on these aspects of child healthcare. In the prepandemic period, virtual visits, leading to a diagnosis and treatment, and referring children to an urgent care or hospital emergency room during regular office hours were rare. During the pandemic, this changed. Significant increased use of telemedicine visits (*p* < 0.0001) and significant decreased office and urgent care visits (*p* < 0.0001) occurred during the pandemic (see Table 1). Telehealth visits peaked the week of April 12, 2020, at 45% of all pediatric visits within the two practices. In-person illness visits gradually returned to year-to-year volumes in August–September 2020 with school opening.

### Well-Child Visits

Early in the pandemic, both pediatric offices limited patient encounters to well-child visits in the first 2 years of life to not miss opportunities for childhood vaccinations. However, some parents were reluctant to bring their children to those visits. There was no significant change in the frequency of healthy visits during the pandemic (although we missed the collection of 48 NP samples at some healthy visits to detect respiratory bacterial colonization rates).

### Changes in Nasopharyngeal Colonization

**Figure 2:**
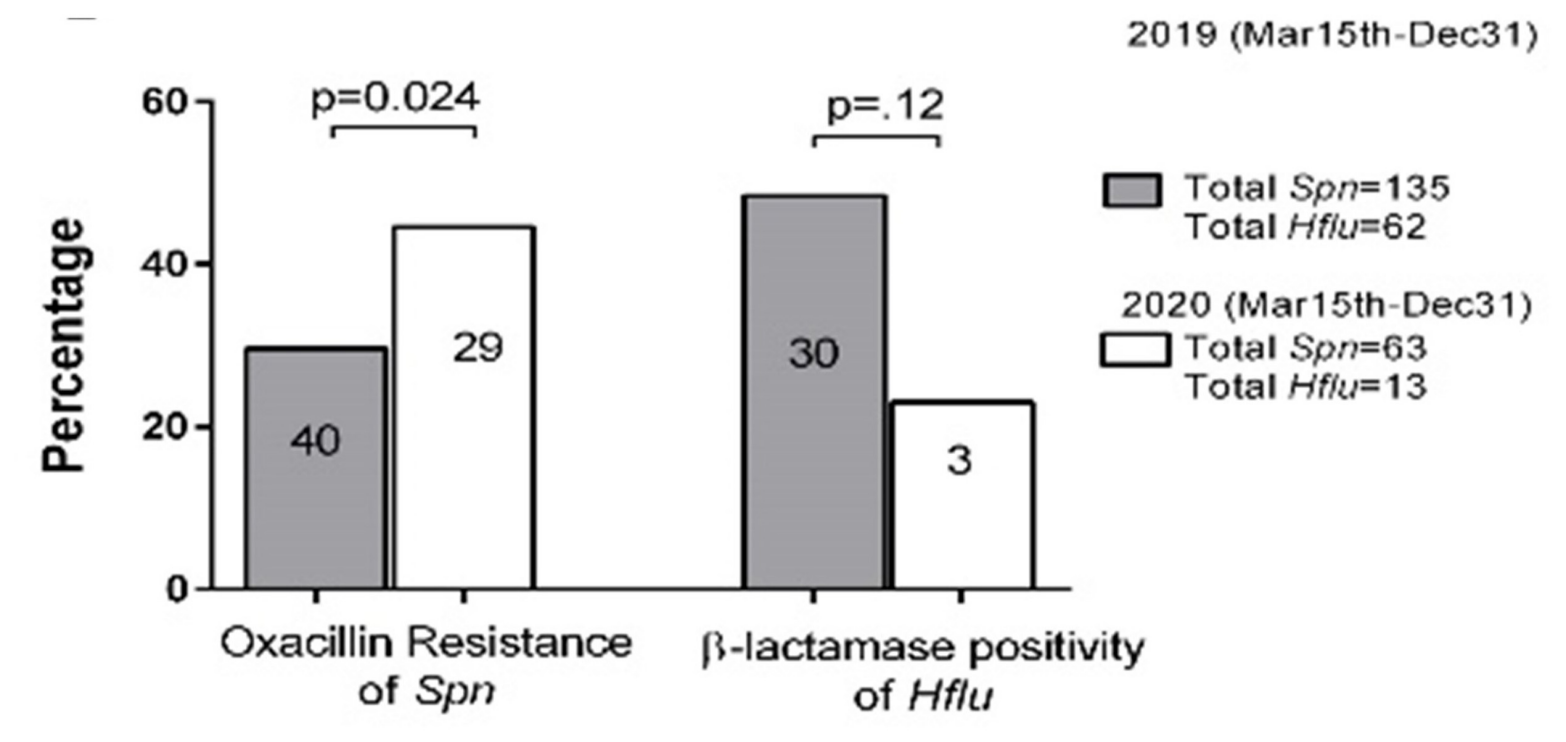
Oxacillin susceptibility and β-lactamase activity comparison in *S. pneumoniae* (*Spn*) and *H. influenzae* (*Hflu*) strains, respectively, before and during the pandemic isolated from NP of children during colonization and AOM cases. All *M. catarrhalis* (*Mcat*) strains were β-lactamase positive (not shown). The number in the bar = oxacillin-resistant and β-lactamase isolates in different times. The *Y*-axis represents the percentage of oxacillin-resistant and β-lactamase positivity among all isolates.

*Spn, Hflu*, and *Mcat* are the three main bacteria that colonize the NP and cause bacterial respiratory infections, including AOM. We compared the isolation rate from the NP of these major respiratory bacteria in the two cohorts. Detection of these bacteria significantly decreased during the pandemic for *Hflu* and *Mcat* (Table 2, *p* < 0.0001) but not for *Spn*. In contrast, isolation of *Spn, Hflu*, and *Mcat* from the NP at onset of AOM, when clinical viral URI was concurrently present, did not differ between the cohorts (Table 2). Oxacillin-resistant *Spn* isolates increased (*p* = 0.009, Figure 2) and β-lactamase-producing *Hflu* isolates decreased during the pandemic. We also compared the isolation rates of specific serotypes of *Spn* isolated from NP and found that isolation of serotype 21 significantly decreased (*p* < 0.05) while isolation of serotypes 15B and 23A significantly increased during the pandemic (*p* < 0.05 and <0.01, respectively; Supplementary Figure 1).

**Table 2:** Comparison of the three main otopathogens in children before and during the pandemic, isolated during healthy and AOM visits, tabulated separately.

**All visits with sample collection**	**497**		**308**		
	**Prepandemic** **March 15–December 31, 2019**		**During pandemic** **March 15–Decembe 31, 2020**		
	**#**	**%**	**#**	**%**	***p*-values**
Healthy visits	389		297		
*Spn*	99	25.2	59	19.8	0.09
*Hflu*	32	8.2	10	3.4	**0.009**
*Mcat*	159	40.8	55	18.5	**<0.0001**
AOM visits	108		11		
*Spn*	36	33.3	4	36.4	ns
*Hflu*	30	27.8	3	27.3	ns
*Mcat*	59	54.6	5	45.5	ns

*ns, not significant (p > 0.05)*.

*P-values marked with bold indicate statistically significance*.

## Discussion

The COVID-19 pandemic modified social habits and contagion control practices. Due to limited public activities during the early quarantine period, in two primary care pediatric practices, during the first 9 months of the COVID-19 pandemic, significant decreases in the frequency of multiple respiratory infections and nasopharyngeal colonization by potential bacterial respiratory pathogens occurred in children age 6–36 months old.

Possible confounding of the illness visit results could have occurred due to changes in the frequency of well-child visits and/or changes in patterns of care from office visits to video telehealth, telephone visits, etc. Nationally, it has been reported that primary preventive services declined among children during the pandemic (24). However, well-child visit frequency did not significantly change during the pandemic in our population because physicians and parents were motivated to keep children up-to-date with childhood vaccinations. Parents often will delay a physician visit if a well-child visit is scheduled in a short time frame from when a child becomes ill. Therefore, understanding the well-child visit frequency was deemed important. Virtual and telephone encounters and referrals to urgent care and hospital emergency rooms did increase during the pandemic as expected because physicians and parents were motivated to keep children out of the physician office, especially at the beginning of the pandemic when so little was known about the contagion. Fortunately, information was available in the EMR allowing capture of alternative visit locations for illness visit care in making comparisons between the cohorts.

Children acquire potentially pathogenic respiratory bacteria by close contact and fomites. The inoculum of *Spn, Hflu*, and *Mcat*, the three main bacteria that colonize the NP and cause bacterial respiratory infections, including AOM is known to increase in the NP during viral URI (25). Therefore, it was not surprising to observe a decrease in the frequency of isolation of the potential bacterial pathogens during the pandemic, coincident with the various isolation and contagion control measures along with less frequent viral URIs. The significant increase in antibiotic resistance of *Spn* isolated from the NP and the significant changes in specific serotypes of *Spn* during the pandemic were unexpected that may reflect relative bacterial virulence and capacity to colonize the NP with more limited inoculum from host to host in the absence of viral URI.

To our knowledge, this is the first study from primary care pediatric practices in the U.S. to analyze the impact on infectious diseases during the first 9 months of the pandemic, including the 6-month time period after the reopening from the first 3 months of lockdown. Our study design capitalized on the collection of prospective data from the beginning of the pandemic lockdown in March 2020 to the end of the year 2020, and for comparison, we used data from a time-matched 9 months just prior to the pandemic. We confined the analysis to children 6–36 months old, when respiratory infections often produce the highest morbidity. One prior study from a primary care network in Massachusetts reported significant decreases in respiratory infectious diseases for children age 0 to 17 years during the first months of the pandemic during lockdown (26). A study in Tennessee that included hospital emergency room, urgent care, primary care, and retail health clinics also reported that respiratory infection diagnoses as well as antibiotic prescription were reduced in the early months of the pandemic (27). Outside the U.S., studies conducted during the first months during lockdown involving pediatric emergency departments in France, Italy, and New Zealand showed significant reductions in emergency visits of children with respiratory disease in a wide age range (28). A reduction in AOM episodes during lockdown was reported from the U.S., Italy, and France from general pediatric (26, 29) and emergency room settings (28, 30, 31). A high rate of improvement in children with chronic otitis media with effusion (OME) was also reported during lockdown (29, 32).

After the first months of lockdown, as France reopened to more social interaction but still required masks, the significant decrease in acute gastroenteritis, common cold, and AOM ED visits bounced back to prepandemic levels (30, 33). Increased infections were not observed in the Massachusetts study, but their follow-up only lasted 5 weeks after the lockdown ended (26). In our study, the incidence of respiratory infections was significantly lower in April 2020, after lockdown, and then at the same level as the prepandemic cohort after reopening. When the new school semester started in September 2020, the lower rates in respiratory infection visits we observed were probably due to ongoing masking, social distancing, and continued restrictions on limits of public gathering.

A lack of immune stimulation due to personal protective measures might induce an “immunity debt” due to reduced circulation of microbial agents, which might grow in susceptible children populations and could negatively affect when the pandemic is under control (34).

## Limitations

During the lockdown, non-essential surgeries, including tympanocentesis, were stopped. After the lockdown, clinicians wore PPE and performing the procedure became technically challenging while wearing a face shield, and parents were disinclined to remain in the clinic for the necessary waiting time to allow topical anesthesia of the tympanic membrane to take effect. Therefore, we were unable to confirm the diagnosis of AOM with bacterial cultures and data collection relied on the EMR. The study was conducted at only two primary care pediatric practices in Upstate NY and that may limit the generalization of observations. We have only few children enrolled (*n* = 215 and *n* = 144) in this analysis and could not provide time series analysis other than that shown in Figure 1. In addition to our small enrolled cohort, we did not have any control visit observation of urinary tract infection although we included gastroenteritis.

## Conclusions

Our study shows an overall reduction in the frequency of respiratory illness visits among children 6–36 months old during the first 9 months of the COVID-19 pandemic. We learned the value of applicability of using technology in the form of virtual visits to render care. The unexpected findings regarding increased NP colonization by antibiotic-resistant bacteria and changes in identified serotypes of *Spn* open new doors of investigation. Perhaps as the pandemic subsides, many of the handwashing and sanitizing practices will remain in place and lead to less frequent illness among children in the future, or the pandemic may produce temporary negative consequences and lead to more frequent respiratory infections because the pool of susceptible children will be larger having been unexposed for over a year (34).

## Data Availability Statement

The raw data supporting the conclusions of this article will be made available by the authors, without undue reservation.

## Ethics Statement

The studies involving human participants were reviewed and approved by Rochester Regional Health Internal Review Board. Written informed consent to participate in this study was provided by the participants' legal guardian/next of kin.

## Author Contributions

RK conceived the study, led the laboratory analysis of all samples tested, and participated in the writing of the manuscript. SS led the clinical enrollment of children into the study and participated in the writing of the manuscript. NF participated in the laboratory analysis of samples tested and participated in the writing of the manuscript. MP conceived the study and study design, oversaw the laboratory analysis of samples and collection of clinical data, participated in the writing of the manuscript, and secured funding to support the work. All authors contributed to the article and approved the submitted version.

## Funding

This study was funded in part (October 2019–September 2020) by CDC (contract #: 75D30119C06842): MP (PI).

## Conflict of Interest

The authors declare that the research was conducted in the absence of any commercial or financial relationships that could be construed as a potential conflict of interest.

## Publisher's Note

All claims expressed in this article are solely those of the authors and do not necessarily represent those of their affiliated organizations, or those of the publisher, the editors and the reviewers. Any product that may be evaluated in this article, or claim that may be made by its manufacturer, is not guaranteed or endorsed by the publisher.
