# Supplementary material for: COVID-19 Pandemic Impact on Respiratory Infectious Diseases in Primary Care Practice in Children
Source: Front Pediatr. 2021 Sep 13;9:722483. doi: 10.3389/fped.2021.722483 (PMC8475492; doi:10.3389/fped.2021.722483)
Supplement: Supplementary file 1 [file Data_Sheet_1.docx]

Recommendations of the NY State Health Department for public health during the study time frame:

On Mar 7, 2020, the New York Governor declared a state of emergency due to the COVID-19 pandemic^1^. The first patient to test positive for SARS CoV2 in Monroe County NY was on Mar 11, 2020^2^. All schools were required to close and non-essential businesses were closed statewide by Mar 22, 2020 as part of “New York State on Pause.”^3^. A mandated public use of masks order for adults and children >2 years of age was enacted Apr 17, 2020^4^. In the Finger Lakes Region of Upstate NY, where the two primary care pediatric practices reside, complete lockdown was partially lifted on May 15, 2020 and further lifted on Jun 26, 2020 in accordance with the Un-PAUSE New York Blue Print Plan^5^. The New York State Office of Child & Family Services released “child care and day camp programs during the COVID-19 public health emergency” guidance around “people, places, and processes” on Jun 26, 2020, with summer day camps being allowed to open on Jun 29, 2020^6^. New York State Education Department released a framework for school reopening on Jul 13, 2020 and NYS Department of Health (DOH) released its final corresponding guidelines on Aug 26, 2020^7, 8^. These guidelines required schools to develop a plan regarding “re-opening of facilities for in-person instruction, monitoring of health conditions, containment of potential transmission of SARS-CoV-2, and closure of school facilities and in-person instruction, if necessitated, by widespread viral transmission.” The NYS DOH released it’s “Pre-K to Gr 12 COVID-19 Toolkit” regarding screening, symptom assessment, testing, and return to school requirements on Sep 2, 2020 which was subsequently revised on Oct 1, 2020 and again Feb 2021^9^. Almost all regional school districts, including those of the Bay Creek and FLMA Pediatrics practice areas, opened to at least hybrid learning models for all students starting Sep 8, 2020. Many schools started with or phased in 4-5 day/week in person instruction for all students. Some districts in the Bay Creek and FLMA Pediatrics practice areas underwent periodic temporary classroom specific or less frequently, all school lockdowns with all virtual learning based on positive SARS-CoV-2 test results among staff, students, and teachers, and/or around holidays, under the direction of the local health department, but not for more than a week or two. All parents were given a choice to have their child(ren) in complete remote virtual learning models and some chose this option. Monroe County (Bay Creek Pediatrics) entered an increasing level of cases designation, “yellow zone” on Nov 9, 2020 due to resurgence of SARS-CoV-2 cases and parts of Monroe County (excluding Bay Creek’s location in Webster, NY) and subsequently entered a higher level of concern designation “orange zone” effective for schools Nov 26, 2020^10, 11^. These two designations required testing a certain number of in-person students and staff on weekly to biweekly intervals. The positivity rate of the testing remained at or below 1% for open Monroe County school districts. Surrounding counties, including Ontario (FLMA Pediatrics) did not enter these higher risk designation zones.

Modifications in medical care due to the pandemic:

Bay Creek and FLMA Pediatrics were in the Rochester Regional Health (RRH) pediatric practice network. On March 6, 2020, RRH coordinated and mobilized efforts for the system’s clinics, which included those in the study. New triage protocols were implemented. This included the introduction of video telehealth and telephone call visits. Patient, staff screening, and safety plans were developed, and personal protective equipment was issued. The week of Mar 16, 2020, the first pediatric COVID-19 community meeting was held to coordinate responses between Rochester area health systems and private pediatric practices. It was decided that in person well visits were to be held only for children 12 months and under. Over subsequent weeks, well visits were expanded to 2 years of age, then 5 years, and then included 10-13 year olds, then to 16 year olds, and ultimately all ages by late May 2020.

Changed practices in Day Care and Schools:

During the “stay at home” phase of the NY State lockdown, day care services were considered an essential business to enable parents who worked as essential workers to go to work^12^. Although day care facilities were not ordered to close, they were limited for families who worked as essential workers in order to prioritize continuous services to the community and limit day care density^13^. Many childcare facilities closed and the remainder operated with decreased capacity. All schools, including pre-K to higher education (Universities) canceled all in person classes and outside school activities and commenced virtual learning, with children and adolescents kept home.

During May and June 2020, reopening of day care facilities occurred gradually based on their enrollment. All children over 2 years old were required to wear a mask while in the facility. Intensified wiping and sanitizing of classrooms/toys and heavily touched surfaces was implemented. Upon arrival, parents were required to answer health check and travel history questions and if any response suggested exposure to SARS-CoV-2, the child was excluded^6, 14^. Temperatures were taken from all children before entry each day and those with fever were excluded until they were fever-free for at least 72 hours. Children with any viral URI symptoms were excluded and it was required that the child had to be symptom-free for at least 72 hours before attendance could resume. Frequent hand washing by care providers and of the hands of children was included in the daily routine. The number of children in day care were reduced as parents elected to keep their children out of the facilities, since many were working from home. Limited SARS-CoV-2 outpatient testing was available and turnaround time was typically > 72 hours from testing to reporting of results to providers and tested persons. Therefore, testing did not affect the stay-at-home rules.

Schools including pre-K to higher education were permitted to reopen beginning in July 2020^15^. However, the majority of schools remained closed because of summer recess. For the school year commencing from September 2020, school operations varied by school district with three options: 1) hybrid (in person classes and virtual), 2) only virtual classes, to reduce the density at school and 3) in person, with all desks kept 6 feet apart, everyone in a mask, no gatherings for lunch or group activities. Almost all regional school districts, including those of the Bay Creek and FLMA Pediatrics practice areas, opened to at least hybrid learning models for elementary students starting Sep 8, 2020. Many schools started with or phased in 4-5 day/week in person instruction for all students. Some districts in the Bay Creek and FLMA Pediatrics practice areas underwent periodic temporary classroom specific or less frequently, all school lockdowns with all virtual learning based on positive SARS-CoV-2 test results among staff, students, or teachers, or around holidays, under the direction of the local health department, but not for more than a week or two. All parents were given a choice to have their child(ren) in complete remote virtual learning models and some chose this option. On each school day, parents were required to answer health check questions regarding SARS-CoV-2 symptoms and students had their temperature taken before entry. Exclusion occurred similar to the day care facilities.

**SARS-CoV-2 in Upstate NY**

One pediatric practice was in Monroe County (Bay Creek) and during the resurgence of the Covid-19 pandemic in the fall, 2020, Monroe County had a sufficiently high SARS-CoV-2 positive test case and hospitalization rate that it teetered on becoming a “red zone” scheduled for return to lockdown for many weeks. In December 2020, the last month of the study, 19% of children tested in the Bay Creek practice were SARS-CoV-2 positive, a very high rate for children. The small town of Geneva, NY (FLMA Pediatrics) in an adjacent NY county saw an increasing rate of SARS-CoV-2 positive cases and hospitalizations during the Fall, 2020, pandemic resurgence in NY State. While the public was aware of Covid-19-related cases and hospitalizations in every County from daily public media (television, radio and newspapers), the number of visits for young children was significantly lower than the time-matched pre-pandemic study period.

References:

1. State of New York. Declaring a Disaster Emergency in the state of New York. <https://www.governor.ny.gov/news/no-202-declaring-disaster-emergency-state-new-york>. Published 2020. Accessed 03/17, 2021.

2. WROC. COVID-19 coronavirus arrives in Monroe County, local events begin to cancel. CORONAVIRUS. <https://www.rochesterfirst.com/coronavirus/1st-confirmed-case-of-covid-19-coronavirus-in-monroe-county/>. Published 2020. Updated 03/13/2020. Accessed 03/17, 2021.

3. State of New York. New York State on PAUSE. Novel Coronavirus (COVID-19). <https://coronavirus.health.ny.gov/new-york-state-pause>. Published 2020. Accessed 03/17, 2021.

4. State of New York. Continuing Temporary Suspension and Modification of Laws Relating to the Disaster Emergency. <https://www.governor.ny.gov/sites/governor.ny.gov/files/atoms/files/EO_202.17.pdf>. Published 2020. Accessed 03/17, 2021.

5. State of New York. Amid Ongoing COVID-19 Pandemic, Governor Cuomo Outlines Blueprint to Un-PAUSE New York. CORONAVIRUS. <https://www.governor.ny.gov/news/amid-ongoing-covid-19-pandemic-governor-cuomo-outlines-blueprint-un-pause-new-york>. Published 2020. Accessed 03/17, 2021.

6. State of New York DoH. Interim Guidance for Child Care and Day Camp Programs During the COVID-19 Public Health Emergency. <https://ocfs.ny.gov/main/news/2020/COVID-2020Jun26-Day-Camp-Detailed-Guidelines.pdf>. Published 2020. Accessed 03/17, 2021.

7. Department NYSE. State Education Department Presents Framework of Guidance to Reopen New York State Schools. <http://www.nysed.gov/news/2020/state-education-department-presents-framework-guidance-reopen-new-york-state-schools>. Published 2020. Accessed 03/17, 2021.

8. State of New York DoH. Interim Guidance for In-person Instruction at Pre-K to Grade 12 Schools during the COVID-19 Public Health Emergency. <https://www.governor.ny.gov/sites/governor.ny.gov/files/atoms/files/Pre-K_to_Grade_12_Schools_MasterGuidance.pdf>. Published 2020. Accessed 03/17, 2021.

9. State of New York DoH. Pre-K to Gr 12 COVID-19 Toolkit. <https://coronavirus.health.ny.gov/system/files/documents/2021/02/nysdoh_prekgr12_toolkit_update-020121.pdf>. Published 2021.

10. WHECTV. Parts of Monroe County in Yellow Zone due to rising COVID-19 cases. <https://www.whec.com/coronavirus/cuomo-announces-covid-19-yellow-zones-in-parts-of-monroe-county/5919378/>. Published 2020. Accessed 03/17, 2021.

11. WROC. Gov. Cuomo: COVID-19 orange zone for parts of Monroe County. <https://www.rochesterfirst.com/new-york-state/watch-live-gov-cuomo-to-provide-update-on-covid-19-in-new-york-state/>. Published 2020. Updated 11/24/2020. Accessed 03/17, 2021.

12. State of New York DoH. Day Care Providers FAQs <https://ocfs.ny.gov/main/news/covid-19/guidance.php>. Published 2020. Accessed 03/17, 2021.

13. State of New York OoCaFS. COVID-19 Guidance to Child Care Providers. <https://www.ocfs.ny.gov/main/policies/external/ocfs_2020/INF/20-OCFS-INF-09-COVID.pdf>. Published 2020. Accessed 03/17, 2021.

14. State of New York OoCaFS. Letter to childcare provider. <https://ocfs.ny.gov/main/childcare/letterstoproviders/2020/Dear-Provider-2020Mar11-Corona-Virus.pdf>. Published 2020. Accessed 03/17, 2021.

15. State of New York. Phase Four Industries. NY forward. <https://forward.ny.gov/phase-four-industries>. Published 2020. Accessed 03/17, 2021.

Supplemental table S1: Demographic of children comparison in pre-pandemic vs during pandemic.

|  | Pre-pandemic | Post-pandemic |  |
| --- | --- | --- | --- |
| Variable Children # | 215 | 144 | p-value |
| Gender |  |  | ns |
| *Female* | 82 (38.1%) | 61(42.4%) |  |
| *Male* | 120(55.8%) | 75 (52.1%) |  |
| Race |  |  | ns |
| *White* | 163 (75.8%) | 109 (75.7%) |  |
| *African American* | 7 (3.3%) | 5 (3.5%) |  |
| *Hispanic* | 9 (4.2%) | 7 (4.9%) |  |
| *Asian* | 2 (0.9%) | 1 (0.7%) |  |
| *Mix* | 34 (15.8%) | 22 (15.3%) |  |
| Siblings |  |  | ns |
| *Yes* | 153 (71.2%) | 102 (70.8%) |  |
| *No* | 62 (28.8%) | 42 (29.2%) |  |
| Daycare |  |  | ns |
| *Yes* | 75 (34.9%) | 54 (37.5%) |  |
| *No* | 140 (65.1%) | 89 (61.8%) |  |
| Breast feeding |  |  | ns |
| *Exclusively (100%)* | 41 (19.1%) | 34 (23.6%) |  |
| *Most of the time (> 50%)* | 36 (16.7%) | 19 (13.2%) |  |
| *Some of the time (< 50%)* | 75 (34.9%) | 40 (27.8%) |  |
| *Very little/none (0 – 5%)* | 63 (29.3%) | 51 (35.4%) |  |
| Smoker in home |  |  | ns |
| *Yes* | 47 (21.9%) | 31 (21.5%) |  |
| *No* | 168 (78.1%) | 113 (78.5%) |  |
| Delivery type |  |  | ns |
| *C-section* | 63 (29.3%) | 46 (31.9%) |  |
| Infant received antibiotic since birth | 110 (51.2%) | 69 (47.9%) | ns |
| Mother flu vaccination | 166 (77.2%) | 107 (74.3%) | ns |
| Ear infection history in family (Parents/siblings) |  |  | ns |
| *No* | 83 (38.6%) | 56 (38.9%) |  |
| *Yes* | 132 (61.4%) | 88 (61.1%) |  |

Ns means not significant (p-value >0.1)
